# Neuropsychiatric Symptoms in Patients with Aortic Aneurysms

**DOI:** 10.1371/journal.pone.0022632

**Published:** 2011-07-22

**Authors:** Bernhard T. Baune, Steven J. Unwin, Frances Quirk, Jonathan Golledge

**Affiliations:** 1 Discipline of Psychiatry, University of Adelaide, Adelaide, South Australia, Australia; 2 Discipline of Psychiatry, James Cook University, Townsville, Queensland, Australia; 3 Vascular Biology Unit, James Cook University, Townsville, Queensland, Australia; The University of Melbourne, Australia

## Abstract

**Background:**

Emerging evidence suggests that vascular disease confers vulnerability to a late-onset of depressive illness and the impairment of specific cognitive functions, most notably in the domains of memory storage and retrieval. Lower limb athero-thrombosis and abdominal aortic aneurysm (AAA) have both been previously associated with neuropsychiatric symptoms possibly due to associated intracerebral vascular disease or systemic inflammation, hence suggesting that these illnesses may be regarded as models to investigate the vascular genesis of neuropsychiatric symptoms. The aim of this study was to compare neuropsychiatric symptoms such as depression, anxiety and a variety of cognitive domains in patients who had symptoms of peripheral athero-thrombosis (intermittent claudication) and those who had an asymptomatic abdominal aortic aneurysm AAA.

**Methodology/Principal Findings:**

In a cross-sectional study, 26 participants with either intermittent claudication or AAA were assessed using a detailed neuropsychiatric assessment battery for various cognitive domains and depression and anxiety symptoms (Hamilton Depression and Anxiety Scales). Student t test and linear regression analyses were applied to compare neuropsychiatric symptoms between patient groups. AAA participants showed greater levels of cognitive impairment in the domains of immediate and delayed memory as compared to patients who had intermittent claudication. Cognitive dysfunction was best predicted by increasing aortic diameter. CRP was positively related to AAA diameter, but not to cognitive function. AAA and aortic diameter in particular were associated with cognitive dysfunction in this study.

**Conclusions/Significance:**

AAA patients are at a higher risk for cognitive impairment than intermittent claudication patients. Validation of this finding is required in a larger study, but if confirmed could suggest that systemic factors peculiar to AAA may impact on cognitive function.

## Introduction

New evidence is emerging suggesting that vascular disease confers vulnerability to a late-onset of depressive illness and the impairment of specific cognitive functions, most notably in the domains of memory storage and retrieval [1], [2]. The relationship between vascular disease and cognitive functions is pathophysiologically and clinically complex and the underlying biological mechanisms are not well understood [3].

The term “Vascular Depression” has been used to describe the broad nature of late-life depression resulting from a multitude of potential vascular mechanisms. Consistent with the Vascular Depression Hypothesis, patients with a later onset of depression have greater neuropsychological and neuroradiological abnormalities, increased disability, medical morbidity and mortality [4], [5], [6]. Whilst the vascular depression topic has received much attention in the literature, the mechanisms through which this relationship is mediated are however unclear. For instance, depression can be understood in terms of the consequence of, or as an independent risk factor for vascular disease, which has been overwhelmingly evident in cases of coronary heart disease (CHD). Patients with a history of ischemic heart disease were more likely to suffer from depression than control patients of similar age and social class [7]. Likewise, depression has been found to be a significant risk factor for cardiac mortality in both men and women [8]. In addition, depression has also been more frequently observed in patients after having suffered a stroke [9].

Traditionally, psychological and psychiatric conditions such as stress and depression have been associated with poor immune system function and an increased susceptibility towards infectious disease and illness [10]. One of the oldest and most consistent biological disturbances in depression is the dysregulation of the hypothalamic-pituitary-adrenal (HPA) axis [11], [12]. HPA function is vital to the regulation of glucocorticoid release and impairment of this function has been shown to induce hypercholesterolemia, hypertriglyceridemia, hypertension and impairment of vascular endothelial cells [13]. Some researchers [13] argue that high levels of cortisol are also significantly associated with the severity and progression of CHD. Other research has also highlighted the presence of immunological events occurring in depressed patients [14] suggesting that immune activation may be causal in the onset of depression [15]. The administration of inflammatory cytokine interleukin-1 (IL-1), induces a series of symptoms referred to as ‘sickness behaviour’, which is closely associated with the symptomology of depressive illness [16]. Interestingly, other inflammatory markers such as C-reactive protein (CRP) and interleukin-6 (IL-6) which are typically associated with CHD have been discovered in the blood samples of depressed patients [17].

The same vascular mechanisms which are associated with vascular depression may also increase vulnerability towards cognitive decline and dementia [2], [18]. Hypertension, atrial fibrillation and low levels of high-density lipoprotein are significantly associated with low cognitive performance and subcortical features including Parkinsonism, gait disturbance and impaired executive function [19]. These findings are consistent with more recent evidence suggesting that vascular pathology is closely associated with cognitive impairment [2], [20], [21], [22]. Evidence from immunological studies suggests that pro-inflammatory cytokines (IL-1β, IL-6 and TNF-α) are implicated in the development and progression of cognitive dysfunction [23], [24]. Research on the association between peripheral vascular diseases and mood and cognitive performance is an area which requires more attention in order to explore the mechanisms of this relationship further.

The current investigation aims to explore the relationship between abdominal aortic aneurysms (AAA) and the symptoms of depression and cognitive functioning. Both local and systemic inflammation has been identified in patients with AAA, with increased circulating levels of a range of cytokines previously identified, such as resistin and osteopontin (OPN) [25], [26]. Such circulating cytokines could impact upon cognitive and emotional centres in the brain. We are aware of no published studies in which neuropsychiatric symptoms have been assessed in patients with AAA except around the time of surgery [27]. Research in this area is particularly significant given that the number of patients with AAA appears to be increasing [28]. In addition, the study may also be helpful to improve the understanding of the vascular genesis of neuropsychiatric symptoms.

It is hypothesised that patients with infrarenal AAA have an increased risk towards the symptoms of depression and cognitive impairment in comparison to patients with occlusive atherosclerosis (without AAAs). We chose these two groups of patients since the mechanisms underlying the pathogenesis of abdominal aortic aneurysm (AAA) and occlusive atherosclerosis appears to have a number of distinctions [29]. An example is the negative association of diabetes with AAA but its positive association with occlusive athero-thrombosis [30]. There is also evidence that certain circulating cytokines and biomarkers may be distinct to aortic aneurysm [31]. Based on these differences between aortic aneurysm and athero-thrombosis it was hypothesised that the association with neuropsychiatric symptoms and these diseases may also be distinct. In order to examine this hypothesis we assessed a range of neuropsychiatric symptoms such as depression, anxiety and a variety of cognitive domains in a group of subjects with asymptomatic AAAs and a separate collection of patients who had symptoms of lower limb athero-thrombosis, namely intermittent claudication, but did not have AAAs.

## Methods

### Study Population

Participants in the current study were recruited from around the local Townsville area that had undergone, or were currently receiving treatment for a blood vessel disease in the Vascular Clinic at The Townsville Hospital. Prior to invitation, participants were screened in order to assess research suitability. Only participants with either symptoms of intermittent claudication or an AAA were invited to participate. Ethics approval was provided by the local ethics committees of the Townsville Hospital and James Cook University. Participants gave written informed consent to participate in the study prior to commencing any study procedures. Participants were either contacted at home or were directly approached during consultation hours at the Vascular Clinic. Collection and analysis of blood serum was processed by a commercial pathology laboratory (Sullivan Nicolaides Pathology). Pathology tests included full blood count, fasting lipids, electrolytes and urea. Physician based medical history confirmed that all patients were free of mental retardation or dementia history or other psychiatric disorders such as schizophrenia. Participation in the research was entirely voluntary and no incentives were offered for participation.

### Assessment of cognitive functioning

The cognitive status of participants was assessed using the *Repeatable Battery for the Assessment of Neuropsychological Status* (RBANS) [32]. The RBANS is a brief individually administered tool which measures five domains of cognitive function including attention, language, visuospatial/constructional abilities, and immediate and delayed memory. The RBANS was developed primarily as a neurological assessment tool for dementia in the elderly. However, success for screening neurocognitive status in younger patients has also been highlighted [33]. Patient fatigue is a drawback of many existing forms of neurological testing which are often lengthy in design, and in some instances may exceed 6 hours [34]. Low administration times allow the RBANS to be administered in as little as 20 minutes, making it ideal for use with elderly patients [33].

Normative data is based on a standardised U.S. sample of 540 adults aged 20–89 years. Factors such as age, sex, race/ethnicity, education level and geographic region were evenly weighed in accordance with 1995 Census data. Reliability coefficients for each index were calculated using spilt-half reliability and Spearman-Brown correction. Reliability coefficients calculated for each subtest and age group ranged from .77 to .95 [33]. Content validity of the RBANS is deemed adequate and comparable to similar clinical tests including the WAIS-III, *Boston Naming Test* and verbal fluency tests. Inter-correlation scores between indexes remained at a moderate range of .20–.40, suggesting that each RBANS index is measuring different and distinct cognitive constructs [33].

### Assessment of Depression and Anxiety

Depressive and anxiety symptoms were assessed using the *Hamilton Depression and Anxiety Scales*. The Hamilton scales is a semi-structured interview consisting of 17 items which assess depressed mood, suicide, work and loss of interest, retardation, agitation, gastro-intestinal symptoms, general somatic symptoms, hypochondriasis, insight and weight loss [35]. The Hamilton Scales was one of the first psychometric tools for the assessment of depression [36], and currently remains one of the most widely used clinical measures for depression [37]. Internal consistency and test-retest reliability coefficients were .91 and .96 respectively, indicating suitability for use in clinical applications [37]. Measures of validity were sufficient across scales with correlations between the Hamilton Depressive Inventory, the Beck Depression Scale and the Beck Hopelessness Scale were .93 and .79 respectively [37]. The Hamilton Depressive Inventory also performed well in discriminating between groups of depressed and non-depressed patients. A significant difference (*p*<.0001) was found between depression, anxiety and control groups [37].

### Assessment of AAA, intermittent claudication and other risk factors

Patients recruited to the study had either an AAA or intermittent claudication. Intermittent claudication was diagnosed by a consultant vascular physician based on an appropriate history along with clinical signs of lower limb ischemia and computed tomography angiography (CTA) evidence of occlusive or stenotic peripheral artery disease. AAA was defined as maximal infra-renal abdominal aortic diameter ≥30 mm from axial slices of the CTA. Patients with AAA included in the current study did not have symptoms of intermittent claudication or clinical evidence of impaired lower limb blood supply. Patients with intermittent claudication included in the current study had an AAA excluded by CTA imaging (aortic diameter <30 mm). Hypertension and diabetes were defined by previous history or treatment for these conditions. Cigarette smoking classification was based on smoking history (defined as ever or never smokers). CHD was defined by a history of myocardial infarction, angina or coronary revascularisation. Body mass index (BMI) was calculated as weight(kg)/height(m)^2^. Specific blood tests for this analysis included C-reactive protein (CRP), Haemoglobin (HB g/l), Triglycerides (TAG) and Low Density Lipoprotein (LDL).

### Statistical Analysis

Demographic and clinical data were compared between AAA and intermittent claudication groups using Chi-square test for categorical data and two sample t-test for continuous data (Table 1). We employed univariate analyses for mean comparison across three groups (age, education, marital status) and student t-test for comparison of means of two groups (patient groups, gender, smoking status) as shown in Table 2. In addition, we used student t-test for mean comparisons of individual and summary cognitive test scores from the RBANS across both vascular patient groups (AAA vs IC) (Table 3). Linear regression analysis was used to assess the within-group effects adjusted for age and education (Table 4). Gender was excluded as a covariate on a methodological basis as only 1 female was assessed in the AAA group. All statistical analyses were carried out using SPSS 16.0 for Windows (SPSS, Chicago, IL, USA).

**Table 1:** Basic socio-demographic and clinical characteristics of patients with AAA or Intermittent Claudication.

	*N*	AAAN = 11	ICN = 15	p-value *
**Ages**, N (%)				
Mean, SD43–64	269	72.4, 5.72 (18.2)	67.2, 9.07 (26.9)	0.110.31
65–74	8	4 (36.4)	4 (26.7)	
75–80	9	5 (45.5)	4 (26.7)	
**Gender**, N (%)				
Male	20	10 (90.1)	10 (66.7)	0.15
Female	6	1 (9.09)	5 (33.3)	
**Education**, N (%)				
Mean, SD8 Years	266	9.0, 0.72 (18.2)	9.1, 0.97 (46.7)	0.890.31
9 Years	12	5 (45.5)	5 (33.3)	
10+ Years	8	4 (36.4)	3 (20.0)	
**Marital Status**, N (%)				
Married	17	7 (63.6)	10 (66.7)	0.58
Divorced	4	2 (18.2)	2 (13.3)	
Single#	5	2 (18.2)	3 (20.0)	
**Smoking**, N (%)				0.748
Never smoked	8	4 (36.4)	4 (26.7)	
Ever smoked	18	7 (63.6)	11 (73.3)	
**Diabetes**, N (%) (2 missing)				0.39
Yes	11	4 (36.4)	7 (53.9)	
No	13	7 (63.6)	6 (46.1)	
**Hypertension**, N (%) (2 missing)				0.85
Yes	17	8 (72.7)	9 (69.2)	
No	7	3 (27.3)	4 (30.8)	
**CHD**, N (%) (5 missing)				0.28
Yes	10	6 (60.0)	4 (36.4)	
No	11	4 (40.0)	7 (63.6)	
**BMI (kg/m^2^)**, mean (SD)		29.7 (4.4)	29.8 (4.1)	0.97
**CRP**, mean (SD)		4.2 (2.8)	3.2 (2.5)	0.11

SD denotes standard deviation; AAA denotes abdominal aortic aneurysm; IC denotes intermittent claudication; CHD denotes coronary heart disease; BMI denotes body mass index; CRP denotes C-Reactive Protein; # includes ‘widowed’ participants;

* p-value of Chi-square test for categorical data and two sample t-test for continuous data.

**Table 2:** Neurocognitive and Psychiatric Symptoms across Vascular Sample.

	*N*	RBANSTotal Score(Mean, SD)	Hamilton Depression Score (HAM-D -17)(Mean, SD)	Hamilton Anxiety Score (HAM-A -14)(Mean, SD)
**Ages**				
** 43–64**	9	99.4 (18.1)	5.0 (3.5)	5.1 (2.8)
** 65–74**	8	96.7 (15.5)	4.7 (6.8)	5.0 (5.3)
** 75–80**	9	87.4 (19.5)	3.4 (4.3)	3.2 (3.4)
**Gender**				
** Male**	20	96.8 (17.3)	4.1 (4.7)	4.1 (3.7)
** Female**	6	86.6 (19.5)	5.1 (5.5)	5.3 (4.5)
**Education**				
** 8 Years**	6	90.8 (1.1)	2.3 (1.8)	3.6 (1.9)
** 9 Years**	12	94.4 (2.1)	6.5 (5.9)	5.6 (4.6)
** 10+ Years**	8	97.2 (2.1)	2.7 (3.3)	3.1 (3.5)
**Smoking**				
** Never smoked**	8	106.2 (19.9)	4.4 (5.7)	5.6 (6.7)
** Ever smoked**	18	91.7 (16.8)	4.4 (1.0)	4.1 (3.1)
**Marital Status**				
** Married**	17	95.9 (19.3)	4.8 (5.4)	4.8 (4.5)
** Divorced**	4	97.0 (9.8)	2.7 (3.4)	2.2 (1.8)
** Single#**	5	87.4 (19.3)	4.2 (3.9)	4.6 (1.8)
**Patient Groups**				
** IC**	15	100.8 (18.2)	4.2 (4.1)	4.3 (3.1)
** AAA**	11	85.7 (14.0) *	4.6 (5.8)	4.5 (4.8)

AAA denotes abdominal aortic aneurysm; IC denotes intermittent claudication; RBANS denotes Repeatable Battery for the Assessment of Neuropsychological Status; # includes ‘widowed’ participants:

* p-value derived from univariate analysis for mean comparison of variables with three groups (age, education, marital status) and student t-test for comparison of means of variables with two groups (patient groups, gender): p-value = 0.031.

**Table 3:** RBANS Single Cognitive Test and Summary Scores across Vascular Groups.

	AAA groupN = 11	IC groupN = 15	p-value†
**Age**, mean (SD)	72.3 (5.7)	67.2 (9.0)	n.s.
**Education in years**, mean (SD)	9.0 (.70)	9.1 (.91)	n.s.
**Single test scores**, mean (SD)			
** List Learning**	17.6 (3.9)	23.7 (6.2)	.027
** Story Memory**	9.0 (4.0)	14.8 (5.0)	.010
** Figure Copy**	18.5 (1.2)	18.4 (2.7)	n.s.
** Line Orientation**	15.9 (2.8)	15.0 (5.1)	n.s.
** Picture Naming**	9.8 (.40)	10.0 (0.0)	n.s.
** Semantic Fluency**	22.5 (6.3)	29.0 (6.1)	.022
** Digit Span**	10.8 (2.5)	9.6 (2.2)	n.s.
** Coding**	35.9 (12.3)	43.2 (13.0)	n.s.
** List Recall**	2.4 (2.9)	4.0 (2.3)	n.s.
** List Recognition**	16.6 (2.8)	18.2 (2.0)	n.s.
** Story Recall**	4.1 (3.5)	7.6 (3.1)	.042
** Figure Recall**	10.9 (4.7)	14.5 (4.0)	n.s.
**Summary scores,** mean (SD)			
** Immediate Memory**	67.0 (11.2)	88.3 (21.2)	.009
** Visuospatial/Constructional**	99.9 (15.1)	103.1 (21.9)	n.s.
** Language**	107.9 (13.9)	118.2 (12.3)	n.s.
** Attention**	40.8 (36.1)	45.8 (33.9)	n.s.
** Delayed Memory**	77.5 (21.7)	95.0 (16.9)	.044
**Total Score**	85.7 (14.0)	100.8 (18.2)	.028

AAA denotes abdominal aortic aneurysm; IC denotes intermittent claudication; RBANS denotes Repeatable Battery for the Assessment of Neuropsychological Status; SD denotes standard deviation;

† p-values derived from student t test for mean comparison across two groups (AAA vs IC); n.s. denotes not significant.

**Table 4:** Impact of Serum Biomarkers on Memory Domains of Cognitive Function in Overall Sample.

	Immediate Memory†		Delayed Memory†		Total Score†	
	beta	95% CI	beta	95%CI	beta	95%CI
**Aortic dia.**	−0.52	−12.04 – −0.05*	−0.49	−12.6 – −1.2*	−0.44	−10.9 – −2.9*
**CRP**	−0.05	−2.9 – 2.3	0.09	−2.2 – 3.3	0.17	−1.5 – 3.2
**HB g/l**	0.07	−0.57 – 0.79	0.17	−0.4 – 0.91	0.07	−0.5 – 0.7
**TAG**	−0.25	−0.16 – 4.5	−0.14	−14.5 – 7.70	−0.25	−14.4 – 4.1
**LDL**	−0.24	−12.36 – 3.44	−0.38	−14.7 – 0.70	−0.3	−11.4 – 2.1

† Linear regression adjusted for age and education; beta = standardized beta-coefficient; CI = confidence interval;

*significant at p<0.05;

CRP: C-reactive protein; HB g/l: Haemoglobin g/l; Aortic dia: aortic diameter; TAG: Triglycerides; LDL: low density lipoprotein.

## Results

A total of 26 patients were assessed for depression, anxiety and cognitive functioning. The sample included 20 male (*M* = 70.3 years, *SD* = 6.3 years) and 6 female (*M* = 66.1 years, *SD* = 1.27 years) participants of Caucasian descent. Measure of education was total years of school completed, the sample ranged from 8–11 years (*M* = 9.1years, *SD* = .82 years). The AAA group consisted of 10 males and 1 female whilst the intermittent claudication group had 10 males and 5 females. Sociodemographic and clinical characteristics such as diabetes, hypertension, smoking, CHD, BMI and CRP showed no significant differences between the AAA and intermittent claudication groups (Table 1). Approximately 19% of all participants were classified as depressed (HAM-D score > 8). Interestingly, symptoms of depression and/or anxiety were not significantly associated with age, gender, education, marital status or vascular group, whereas the total cognitive test score showed a significant difference between vascular groups indicating a poorer overall cognitive performance of the AAA group. Summary levels of depression, anxiety and cognitive functioning stratified by age, gender, education, marital status and vascular group are presented in Table 2.

Table 3 presents between-group comparisons of both single cognitive test scores and summary scores. The RBANS total score was compared between the AAA and intermittent claudication patient groups and revealed a significant difference between both groups (*p* = 0.028) with AAA patients performing worse than intermittent claudication patients. AAA group participants generally scored worse on most individual and summary cognitive scores as compared to intermittent claudication participants. Significant differences were noted on tasks involving short and long-term memory (Table 3). Participants in the AAA group also displayed difficulties in domains of language and attention as indicated by the mean scores; however, these differences were not significant.

Linear regression analyses displayed in Table 4 investigated the potential effect of biological variables on the memory domains (immediate and delayed) and the total score which as shown above had demonstrated significant differences between AAA and intermittent claudication patients. As a main result aortic diameter increase was associated with reduced immediate (effect size *r* = 0.51; adjusted R^2^ = 0.24) and delayed memory retention (effect size *r* = 0.47; adjusted R^2^ = 0.21) and overall cognitive functioning (effect size *r* = 0.43; adjusted R^2^ = 0.16) (see Table 4 for details). In contrast, blood serum levels of C-reactive protein, haemoglobin, triglycerides and low density lipoprotein showed no significant association with immediate or delayed memory function, either in the whole sample or in the subgroups of patients with AAA or intermittent claudication. Additional adjustment for CHD, hypertension, smoking and diabetes did not change this association.

In additional analyses we observed in the whole sample, that CRP serum levels were significantly associated with aortic diameter after adjustment for age and education (t = 2.6; p = 0.021) indicating a relationship between aortic development and systemic inflammation, which may indicate an indirect relationship between CRP and memory function in our study; however no direct evidence was found for this assumption.

## Discussion

In this cross-sectional study of vascular patients, we examined if neuropsychiatric and neurocognitive symptoms were associated with AAA presence. We found that overall cognitive function and specifically short-term and delayed memory were associated with AAA. Furthermore aortic diameter was associated with worse cognitive scores in the memory domains. In contrast, no association between depressive or anxiety symptoms and AAA was observed.

The findings of this small study suggest worse cognition function in patients with AAA compared to those with occlusive peripheral artery disease. There are numerous possible explanations for these findings. AAA has been associated with distinct circulating concentrations of biomarkers, including pro-inflammatory cytokines, proteolytic enzymes and thrombus products [31]. Recently for example we demonstrated that patients with AAA have higher plasma concentrations of D-dimer compared to those with intermittent claudication [38]. Such circulating products could cross the blood brain barrier and influence cognitive performance. Indeed an association between circulating thrombus products and cognition has been reported in other cohorts [39], [40]. Pro-inflammatory cytokines are also upregulated in patients with AAA, such as resistin, osteoprotegerin and osteopontin [26], [41], [42]. Such circulating cytokines could also alter central nervous system (CNS) immunological function which is believed to influence memory and learning [43]. It is acknowledged that cytokines produced in the periphery can circulate through the blood stream to other areas of the body including the CNS [44]. Communication with brain neurons can occur via direct penetration of the blood brain barrier, or through activation of the vagus nerve within the abdominal and thoracic cavity, which communicates with neuronal populations in the brainstem [45]. In the current study we measured circulating concentrations of a general marker of systemic inflammation CRP. We found a significant association between CRP serum levels and aortic diameter, as has been previously reported [46], however in this study CRP was not associated with cognitive function. It is possible that specific cytokines, such as osteopontin which are known to modulate chronic inflammation through actions on T cells and macrophages, may be more relevant to cognitive impairment than a general marker of inflammation. However, these biomarkers apart from CRP were not measured in our study, suggesting further studies are required in this area.

Early perspectives on systemic inflammation suggested that the CNS was not susceptible to inflammation or immune activation, and was considered to be mostly unaffected by systemic inflammatory and immune response [47]. A more modern opinion now suggests that the brain controls and regulates many aspects of immune response and activation. Recent studies of stroke, multiple sclerosis, Alzheimer's and depressive illness suggest there is a strong interconnection between the immune, endocrine, central and autonomic nervous systems [47] [48]. Immunological studies have focused primarily on the action and reaction of cytokines in response to inflammatory stimuli. Studies suggest that cytokines are highly influential and may act either synergistically or antagonistically towards each other [48]. However, by virtue of effect, cytokines may influence or contribute to symptoms of behavioural pathologies. Extensive research [23], [24], [49], [50] has found that pro-inflammatory cytokines including IL-1β, IL-6 and TNF-α are strongly associated with cognitive decline and motor retardation particularly amongst elderly populations. This follows more recent evidence citing the existence of a ‘*Cytokine Model of Cognitive Function’*. The model proposes that pro-inflammatory cytokines play an intimate role in the molecular and cellular mechanisms sub-serving learning, memory and cognition [43]. The model provides a basis from which to explore complex neuropsychiatric conditions such as cognitive decline, dementia and depression resulting from cytokine activation.

It is possible that cytokines, such as resistin and OPN, which are upregulated in AAA, may also exert their effects on pro-inflammatory cytokines (IL-1β, IL-6, IL-8 and TNF-α) either directly in the CNS, or through a process of peripheral inflammation. It is not yet clear if resistin and OPN are capable of crossing the blood brain barrier; however evidence suggests that it may indeed be possible given the small physical profile of the molecules [51], [52].

Another possible reason for the association between cognition and AAA identified in the current study could be associated intracerebral vascular disease. Intracerebral vascular disease is one of the commonest causes of dementia. It is possible that the patients with AAA we studied could have more severe vascular pathology, including intracerebral disease, than those with intermittent claudication. We did not image the brains of patients in this study making further assessment of this impossible.

### Limitations

The findings from this study should be interpreted in light of methodological limitations particularly in relation to the size and representativeness of the sample. The predictive power of the findings must also be received with some caution. Statistical error must be considered with any research, however, studies with small samples are particularly vulnerable to sampling errors leaving uncertainty about the negative results on depression in this study.

Another limitation which should be considered is the role of gender. Gender amongst age and education is generally acknowledged as a mediating factor in cognitive function [53]. Whilst gender effects were still present in the current findings, females were underrepresented particularly in the AAA group. Future research should focus on achieving more equal gender samples in order to thoroughly examine the differences. Similarly, ethnicity and socio-economic status was limited by patient availability to Caucasians living in lower socio-economic status areas of Townsville. The ability to generalise these results to more diverse cultural backgrounds and other socio-economic groups is reduced and should also be considered for future research. Although in this study, smoking, diabetes, hypertension and BMI showed no significant differences across both vascular groups (AAA vs IC), future studies should consider these and additional factors such as alcohol consumption and severity of atherosclerotic disease as potential confounders.

Further study in this area would be to first replicate the current findings using a larger sample of participants with a greater emphasis on gender and socio-economic status. Whilst the current study has identified observable cognitive differences between genders, the sample remains too small to make any broad generalisations to the general population. Another aspect which was not examined in this study is the relationship between specific cytokines and cognitive function in patients with AAA. In particular, systemic inflammatory markers resistin and osteopontin have been implicated in the pathogenesis of AAA, however, their role in cognitive functioning is currently unknown. Further research needs to closely examine this potential relationship.

In conclusion, this study suggests that AAA and aortic diameter are associated with cognitive performance. Systemic inflammatory and vascular changes related to AAA could account for this association, however further studies are required to examine this.
